# Protocol for non-invasive tumor monitoring and diagnosis based on interpretable deep learning

**DOI:** 10.1016/j.xpro.2026.104358

**Published:** 2026-02-06

**Authors:** Zhenbo Yuan, Yuli Yan, Youpeng Yang, Xin Li

**Affiliations:** 1 School of Medicine, Shenzhen Campus of Sun Yat-sen University, Shenzhen, China

**Keywords:** Bioinformatics, Sequence analysis, Cancer

## Abstract

Tumor-specific DNA methylation profiling in plasma cell-free DNA (cfDNA) offers a promising approach for non-invasive tumor detection. Here, we present a protocol that uses Oncoder, an interpretable deep-learning-based framework, to monitor treatment response by tracking dynamic changes in tumor-specific DNA methylation signals in patient plasma cfDNA. We describe steps for data preparation, performing differential methylation analysis, training Oncoder, and interpreting the model’s outputs. This protocol is versatile and adaptable to various data types and application scenarios.

For complete details on the use and execution of this protocol, please refer to Yang et al.^1^

## Highlights

• A protocol for using Oncoder to predict tumor fractions from cfDNA methylation data
• Instructions for differential DNA methylation analysis
• Steps for training the Oncoder model using tumor-specific DNA methylation markers
• Statistical comparison of dynamics in tumor fractions following drug therapy

## Abstract (editor highlights)

Publisher’s note: Undertaking any experimental protocol requires adherence to local institutional guidelines for laboratory safety and ethics.

## Graphical abstract

**Figure:**
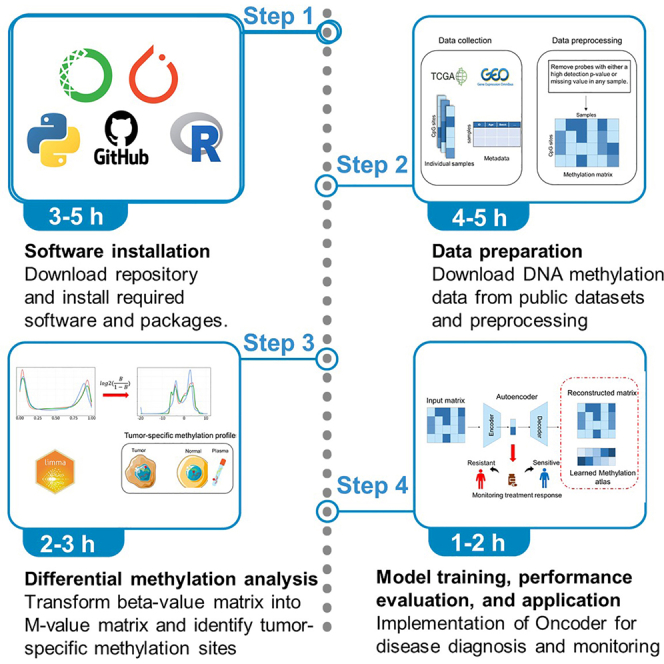

## Before you begin

The detection of circulating tumor DNA (ctDNA) within cell-free DNA (cfDNA) has shown great potential for cancer diagnosis.^2^^,^^3^ However, a major hurdle in using ctDNA as a tumor biomarker is accurately distinguishing the minute fraction of ctDNA from vast background of cfDNA. DNA methylation profiling represents a promising strategy, as tumor-derived ctDNA typically exhibits methylation patterns distinct from those of background cfDNA.^4^ Moreover, DNA methylation profiling is characterized by high stability and broad genomic coverage, offering clear-cut advantages over other biomarkers, such as tumor-specific mutations and chromosomal aberrations.^5^^,^^6^^,^^7^^,^^8^^,^^9^

Here, we describe a protocol that uses Oncoder, an autoencoder-based framework, designed to automatically learn methylation patterns from a reference methylation atlas for predicting tumor fractions in plasma cfDNA. To demonstrate its utility, we leverage Oncoder to monitor the dynamics of tumor fraction in plasma cfDNA across 33 prostate cancer patients during abiraterone acetate (AA) therapy. As treatment with AA is known to largely eliminate non-resistant prostate cancer cells,^10^ this case study presents an illustrative biological scenario.

### Innovation

Various deconvolution methods have been developed to estimate tumor fractions from cfDNA methylation profiles. Traditional approaches, such as Non-negative Least Squares (NNLS),^11^^,^^12^ rely on a comprehensive reference methylation atlas as a basis for convolution target. However, these methods are often limited by the substantial cost of generating high-quality reference and their suboptimal predictive accuracy. Semi-reference-free deconvolution (SRFD) methods, including NMF,^13^^,^^14^ circumvent the need for an explicit reference, but often lack intuitive interpretability, hindering their clinical adoption. Oncoder trains its deconvolution capability using simulated data mixtures of tumor tissue and plasma samples at predefined proportions. The model offers intuitive interpretability: its latent space represents the proportions of normal and tumor fractions, while the decoder’s weight matrix reveals tumor-specific methylation patterns. Thus, Oncoder overcomes the high costs of reference acquisition and achieves a 30% reduction in prediction error and the highest prediction correlation among comparable methods.

### Necessary operating system and hardware

**Timing: 1–2 h**

This section includes the minimal hardware, operating system and software requirements.

1. Install Ubuntu 16.04 or greater unless such an operating system is already available. The minimum requirement for the computer hardware, operating system, and software recommendations:

Ubuntu OS ≥ 16.04 LTS, minimum RAM 64 GB, Python version ≥ 3.6, pip version ≥ 19.0 and R version ≥ 3.5.

### Install required software and libraries

**Timing: 2–3 h**

This section includes the setup of the computational environment, which involves installing all required software, Python packages, and R libraries within a dedicated conda environment.

***Note:*** Conda is a powerful tool to manage multiple projects, particularly when specific versions of Python and R are required. It enables users to create self-contained environments and specify the version of dependencies.

2. Install Anaconda and Git.
  a. Check if Anaconda is available by running the following command in the virtual terminal:
    >conda –version
    If it is not available, install the appropriate version for your operating system by following the installation guide at https://www.anaconda.com/download.
  b. Check if git is installed:

>git –version

If it is not available, install the appropriate version for your operating system by following the installation guide at https://git-scm.com/book/en/v2/Getting-Started-Installing-Git.

3. Set up a python virtual environment and download the repository.
  a. Create a new conda environment for Oncoder:
    >conda create --name Oncoder python=3.13
    >conda activate Oncoder
  b. Clone the Oncoder repository, which contains all necessary scripts:
    >git clonehttps://github.com/yyp1999/Oncoder.git
    >cd Oncoder
  c. Install the required python libraries from the requirements.txt file:

>pip install -r requirements.txt

4. Set up an R virtual environment.
  a. Create and activate a new conda environment for R analysis:
    >conda create --name <R ENV>
    >conda activate <R ENV>
    >conda install -c conda-forge r-base=4.4.1
  b. Install the required packages.

>R

>install.packages("BiocManager")

>install.packages(c("R.utils", "knitr", "stringr", "caret","pheatmap"))

>BiocManager::install(c("GEOquery", "limma"))

>quit()

**CRITICAL:** Before installing GEOquery, please ensure you have the following dependencies installed in your conda environment. If not, please run the command below:

>conda install libxml2 openssl libcurl zlib

**CRITICAL:** Before proceeding to the next step, please ensure all packages are properly installed (troubleshooting 1).

## Key resources table

**Table:** 

REAGENT or RESOURCE	SOURCE	IDENTIFIER
**Deposited data**		

The DNA methylation profiles of 484 primary prostate tumor samples and 49 adjacent non-tumor samples	TCGA	https://portal.gdc.cancer.gov/
The blood cfDNA methylation profiles of 31 prostate cancer samples	Gordevičius et al.^10^	https://www.ncbi.nlm.nih.gov/geo/query/acc.cgi?acc=GSE108462
The blood cfDNA methylation profiles of 656 normal samples.	Hannum et al.^15^	https://www.ncbi.nlm.nih.gov/geo/query/acc.cgi?acc=GSE40279
Code availability	Present study	https://github.com/yyp1999/Oncoder.git

**Software and algorithms**		

Python3	Python v.3.13.7	https://www.python.org/
Matplotlib	Matplotlib v.3.10.6	https://matplotlib.org/
Numpy	NumPy v.2.3.3	https://numpy.org/
PyTorch	PyTorch v.2.8.0	https://pytorch.org/
Pandas	Pandas v.2.3.3	https://pandas.pydata.org/
SciPy	SciPy v.1.16.2	https://scipy.org/
R	R v.4.4.1	https://www.r-project.org/
BiocManager	BiocManager v1.30.26	https://www.bioconductor.org/
GEOquery	GEOquery v.2.74.0	https://www.bioconductor.org/packages/release/bioc/html/GEOquery.html
Limma	Limma^16^ v.3.62.2	https://bioinf.wehi.edu.au/limma/
ggplot2	ggplot2 v.4.0.0	https://ggplot2.tidyverse.org/

**Other**		

Intel(R) Core(TM) i9-10980XE CPU @ 3.00GHz	Intel	https://www.intel.com/content/www/us/en/homepage.html
GNU/Linux Ubuntu 22.04.5 LTS	Canonical Ltd.	https://ubuntu.com/
NVIDIA GeForce RTX 3090 ∗ 3	NVIDIA	https://www.nvidia.com/en-gb/

## Step-by-step method details

**Figure 1:**
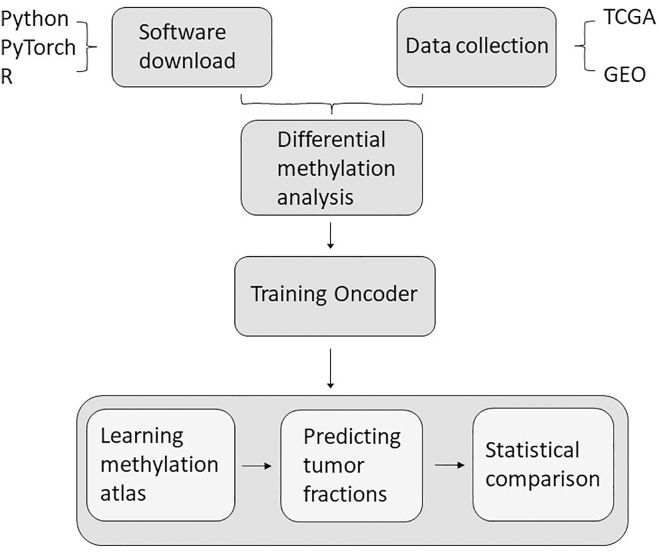
The workflow schematic of this protocol

The workflow schematic of this protocol is illustrated in Figure 1.

### Data collection and preprocessing

**Timing: 4–5 h**

This section details the steps for downloading and preprocessing all required files. Specifically, these files include DNA methylation beta-value matrices derived from prostate cancer tumor and adjacent normal tissues, as well as plasma cell-free methylation matrices from healthy donors and prostate cancer patients classified into AA-resistant and AA-sensitive groups. The corresponding clinical metadata are also provided.

1. Download the tissue-derived DNA methylation files for 484 primary prostate tumor and 49 adjacent normal tissues from The Cancer Genome Atlas (TCGA) database.
  a. Navigate to the GDC Data Portal https://portal.gdc.cancer.gov/.
  b. In the “Cohort Builder” TAB, select “TCGA-PRAD” from the “Project” list.
  c. In the “Disease type” facet, select “acinar cell neoplasms” and “adenomas and adenocarcinomas”.
  d. Switch to “Repository” TAB. Under the “Data Type” filter, select “Methylation Beta Value”.
  e. Click “Manifest” to download the manifest file.
  f. Use the tcga_downloader.py script from DataMiner to automatically download the metadata and the txt files for each sample (troubleshooting 2):
    >git clonehttps://github.com/vappiah/DataMiner.git.
    >cd DataMiner
    >python
    >from tcga_downloader import ∗
    >ids=get_ids('/path/to/your/Manifest')
    >payload=prepare_payload(ids,data_type='Methylation Beta Value')
    >metadata=get_metadata(payload)
    >download_data(metadata,sep='\t',outdir='PRAD')
    >quit
    ***Note:*** Executing the above code will generate a metadata file containing detailed patient information, and a ‘PRAD’ folder where the methylation data is automatically sorted into subfolders by tissue source.
  g. Merge all the txt files from the “Primary Tumor” and “Solid Tissue Normal” folders into two separate matrices.
    >conda activate <R ENV>
    >R
    >metadata <- read.table("/path/to/Metadata.tsv",sep = '\t',header = TRUE)
    >file_paths_tumor <- list.files(path = "/path/to/PRAD/Primary Tumor", pattern = "∗.txt", full.names = TRUE)
    >tumor <- lapply(file_paths_tumor, function(x){read.table(x, header = FALSE, sep = "\t",row.names = 1)})
    >tumor <- do.call(cbind, tumor)
    >colnames(tumor) <-metadata$cases.0.submitter_id[match(basename(file_paths_tumor), metadata$file_name)]
    >file_paths_normal <- list.files(path = "./PRAD/Solid Tissue Normal", pattern = "∗.txt", full.names = TRUE)
    >normal <- lapply(file_paths_normal, function(x){read.table(x, header = FALSE, sep = "\t",row.names = 1)})
    >normal <- do.call(cbind, normal)
    >colnames(normal) <- metadata$cases.0.submitter_id[match(basename(file_paths_normal), metadata$file_name)]
  h. Save the results.

>write.table(tumor,"./tumor_PRAD.tsv",sep = "\t",quote = FALSE)

>write.table(normal, "./normal_PRAD.tsv",sep = "\t",quote = FALSE)

***Note:*** DNA methylation data from the TCGA-PRAD cohort is profiled using the Illumina HumanMethylation450 BeadChip, which covers approximately 450,000 CpG sites across different genomic regions. In the preprocessed matrices, rows correspond to CpG probes and columns represent individual samples. Each entry in the matrix is a beta-value, which indicates methylation level at a specific CpG site.

2. Download the required DNA methylation files from the Gene Expression Omnibus (GEO) database.
  ***Note:*** In the current study, we employ the datasets GSE108462 and GSE40279. Specifically, GSE40279 consists of plasma cfDNA methylation profiles obtained from 656 healthy individuals (aged 19 to 101 years). For GSE108462, a total of 181 cfDNA methylation samples (comprising 120 unique samples and 61 technical replicates) were collected from 33 patients undergoing AA treatment. These samples were obtained at baseline (before starting AA) and on subsequent checkups.
  a. Use GEOquery package to load the Series Matrix Files of GSE108462 and GSE40279 (troubleshooting 3).
    >library(GEOquery)
    >geoMat_plasma <- getGEO("GSE40279")[[1]]
    >geoMat_ctProstate <- getGEO("GSE108462")[[1]]
  b. Extract and preprocess the methylation matrix and corresponding metadata of dataset GSE40279.
    >normal_plasma <- exprs(geoMat_plasma)
    >metadata_plasma <- pData(geoMat_plasma)
    >metadata_plasma <- metadata_plasma[, c("title", "geo_accession")]
    >metadata_plasma <-metadata_plasma[match(metadata_plasma$geo_accession, colnames(normal_plasma)),]
  c. Download the “Unnormalised_signal.csv” file from the supplementary files of GSE108462 and read it into R session.
    >library(data.table)
    >ctProstate <- fread("/path/to/GSE108462_Unnormalised_signal.csv.gz")
    >ctProstate <- as.data.frame(ctProstate)
    >rownames(ctProstate) <- ctProstate$ID_REF
    >ctProstate <- ctProstate[,-1]
  d. Extract the columns for “Detection Pval”, “Unmethylated Signal”, and “Methylated Signal” for all probes and calculate the methylation beta-values.
    >ctProstate_pvalue <- ctProstate[,grepl('Detection Pval', colnames(ctProstate))]
    >probes_to_keep <- which(! rowSums(ctProstate_pvalue >= 0.01) >= 1)
    >ctProstate_Unmethylated <- ctProstate[,grepl('Unmethylated Signal', colnames(ctProstate))]
    >ctProstate_Methylated <- ctProstate[,grepl('Methylated Signal', colnames(ctProstate))]
    >ctProstate_Unmethylated <- ctProstate_Unmethylated[probes_to_keep,]
    >ctProstate_Methylated <- ctProstate_Methylated[probes_to_keep,]
    >ctProstate_norm <- ctProstate_Methylated / (ctProstate_Methylated + ctProstate_Unmethylated + 100)
    ***Note:*** Probes exhibiting a high detection p-value (p ≥ 0.01, indicating that the signal is indistinguishable from background noise) in any sample are discarded from the downstream analysis.
  e. Subset the patients’ metadata based on phenotype.
    >library(dplyr)
    >metadata_ctProstate <- pData(geoMat_ctProstate) %>% select(title, geo_accession, group = characteristics_ch1, tissue = characteristics_ch1.1, ID = characteristics_ch1.3, phenotype = characteristics_ch1.11, timepoint = characteristics_ch1.9, batch = `cohort:ch1`) %>% mutate(across(c(group, tissue, ID, phenotype, timepoint), ∼ sub("ˆ(diagnosis|tissue|individual id|phenotype|visit time point): ", "", .)), phenotype = sub(" to AA treatment", "", phenotype), timepoint = as.numeric(timepoint))
    >colnames(ctProstate_norm) <- metadata_ctProstate$geo_accession
    >filter_and_sort <- function(data, pheno_type) {data[data$phenotype == pheno_type, ] %>% arrange(ID, timepoint)}
    >metadata_ctProstate_Resistant <- filter_and_sort(metadata_ctProstate, 'Resistant')
    >metadata_ctProstate_Sensitive <- filter_and_sort(metadata_ctProstate, 'Sensitive')
  f. Split matrices according to the phenotype, and save all resulting matrices.

>write.table(normal_plasma ,file = "normal_plasma.tsv",sep = "\t",quote = FALSE)

>write.table(metadata_plasma,"metadata_normal_plasma.tsv",sep = "\t",quote = FALSE)

>write.table(ctProstate_norm[,metadata_ctProstate_Resistant$geo_accession],"ctProstate_Resistant.tsv",sep = "\t",quote = FALSE)

>write.table(ctProstate_norm[,metadata_ctProstate_Sensitive$geo_accession],"ctProstate_Sensitive.tsv",sep = "\t",quote = FALSE)

>write.table(metadata_ctProstate_Resistant,"metadata_ctProstate_Resistant.tsv",sep = "\t",quote = FALSE)

>write.table(metadata_ctProstate_Sensitive,"metadata_ctProstate_Sensitive.tsv",sep = "\t",quote = FALSE)

>quit()

**CRITICAL:** After completing Step 1 and Step 2, your current working directory is expected to contain the beta-value matrices for tumor tissue, adjacent normal tissue, normal plasma, and AA-resistant and -sensitive patients, as well as the metadata files for the normal plasma and AA-resistant and -sensitive patient groups.

### Differential methylation analysis

**Timing: 2–3 h**

This section includes the details on identifying tumor-specific differentially methylated positions (DMPs). We perform differential analysis using M-values to contrast prostate tumor tissues against adjacent normal tissues and healthy plasma samples. M-values are derived from beta-values via logit transformation and offer better statistical properties. These identified sites are then used as input features for training the model.

3. Activate conda environment and load the required packages.

>conda activate <R ENV>

>R

>library(knitr)

>library(limma)

>library(stringr)

>library(ggplot2)

>library(tidyr)

>library(dplyr)

>library(tibble)

>library(pheatmap)

>library(RColorBrewer)

4. Load all DNA methylation matrices preprocessed in the first two steps into the R session.

>rm(list=ls())

>matrix_names<-c("tumor_PRAD.tsv","normal_PRAD.tsv", "normal_plasma.tsv","ctProstate_Resistant.tsv","ctProstate_Sensitive.tsv")

>matrix_list <- lapply(martix_names,function(x){read.table(paste0(x),header = T,sep = "\t",row.names = 1)})

>names(matrix_list) <- sub("\\.tsv$", "", martix_names)

5. Remove methylation probes with missing values in any sample.

>probs_to_keep <- Reduce(intersect, lapply(matrix_list, function(x){rownames(na.omit(x))}))

>matrix_list <- lapply(matrix_list ,function(x) {x[probs_to_keep,]})

6. Combine the methylation matrices of prostate cancer tumor tissues, adjacent normal tissues, and plasma samples.

>beta_matrix <- do.call(cbind, matrix_list[1:3])

7. Add group information as column annotation to the combined matrix.

> colnames(beta_matrix) <- c(paste0('tumor_',1:ncol(matrix_list[[1]])),paste0('normal_',1:ncol(matrix_list[[2]])),paste0('plasma_',1:ncol(matrix_list[[3]])))

>group_sizes <- sapply(matrix_list[1:3], ncol)

>group_names <- names(matrix_list[1:3])

>groups <- factor(rep(group_names, times = group_sizes),levels = group_names)

>group_mapping <- data.frame(sample_id = colnames(beta_matrix),group = groups)

**Figure 2:**
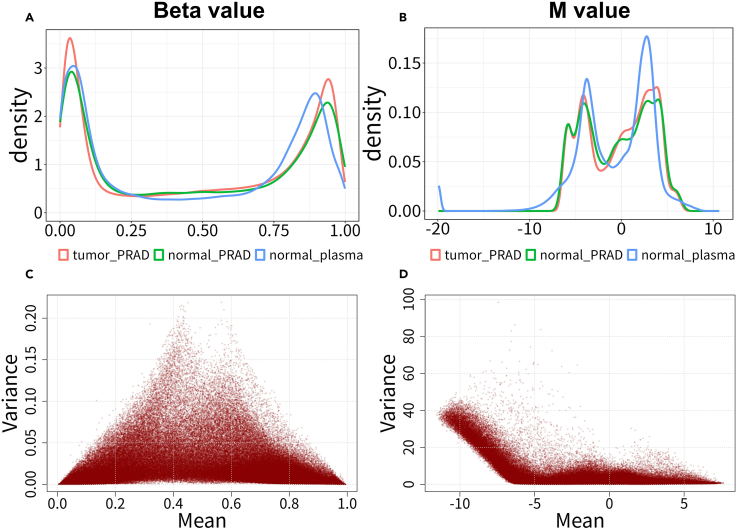
Comparison between beta-values and M-values (A and B) Density plots of (A) beta-values and (B) M-values for the first 2,000 methylation probes across different sample groups. The beta-value ranges from 0 to 1, while the M-value ranges from negative infinity to positive infinity. (C and D) Dot plots of the mean and variance of (C) beta-values and (D) M-values for all probes across different sample groups. The beta-value exhibits low variance near 0 and 1, but high variance near 0.5. In contrast, M-value demonstrates homoscedasticity. Its variance is approximately constant along the X-axis.
  c. Plot the mean-variance relationship of beta-values and M-values (Figures 2C and 2D).

8. Transform beta-value to M-value for subsequent statistical analyses.
  ***Note:*** All methylation matrices obtained from the TCGA and GEO databases consist of beta-values. The beta-value corresponds to the proportion of methylation at this site and has direct biological interpretability. However, it exhibits significant heteroscedasticity outside the intermediate methylation range. In contrast, M-values demonstrate superior performance in differential analysis due to their approximate homoscedasticity.
  a. Utilize the logit function to calculate M-values.
    >M_matrix <- pmin(pmax(as.matrix(beta_matrix),1e-6),1-1e-6) %>% qlogis() / log(2)
  b. Plot the distribution density of beta-values and M-values for the first 2,000 CpG sites(Figures 2A and 2B),
    >plot_methylation_density <- function(mat, filename) {p <- as.data.frame(mat) %>% rownames_to_column("cpg_id") %>% pivot_longer(-cpg_id, names_to = "sample_id", values_to = "value") %>% merge(group_mapping, by = "sample_id") %>%
    ggplot(aes(value, color = group)) + geom_density() + theme_bw() + theme(legend.position = "bottom", plot.title = element_text(hjust = 0.65, size = 20, face = "bold"), axis.title = element_text(size = 16), axis.text = element_text(size = 14), legend.text = element_text(size = 13), legend.title = element_blank(), panel.border = element_rect(color = "black", fill = NA, linewidth = 1))
    ggsave(filename, p, width = 8, height = 6, dpi = 300) }
    >plot_methylation_density(beta_matrix[1:2000,],"Methylation_beta_value.jpg")
    >plot_methylation_density(M_matrix[1:2000,],"Methylation_M_value.jpg")

>mean_beta <- apply(beta_matrix, 1, mean)

>var_beta <- apply(beta_matrix, 1, var)

>mean_M <- apply(M_matrix, 1, mean)

>var_M <- apply(M_matrix, 1, var)

>jpeg("variance.beta_vs_M.jpg", width = 8, height = 6, res = 300 , units = 'in')

>par(mfrow = c(1, 2), mar = c(5, 5, 4, 2))

>plot(mean_beta,var_beta,main = "",xlab = "Mean Beta-value",ylab = "Variance", pch = 16,cex = 0.8, col = "#8B000033")

>grid()

>plot(mean_M, var_M,main = "",xlab = "Mean M-value",ylab = "Variance", pch = 16,cex = 0.8,col = "#8B000033")

>grid()

>dev.off()

9. Identify tumor-specific DMPs using limma package.
  a. Create a design matrix incorporating group information and fit linear models to M-value matrix.
    >design <- model.matrix(∼0+groups)
    >colnames(design) <- levels(groups)
    >fit <- lmFit(M_matrix, design)
  b. Define comparisons between “Prostate tumors vs adjacent normal tissues” and “Adjacent normal tissues vs normal plasma samples”, and apply empirical Bayes moderation to improve the stability and power of the statistical tests.
    >contMatrix <- makeContrasts(tumor_PRAD - normal_PRAD, normal_PRAD -normal_plasma, levels=design)
    >fit2 <- contrasts.fit(fit, contMatrix)
    >fit2 <- eBayes(fit2)
  c. Extract the differential analysis results.
    >tumor_results <- topTable(fit2, number = Inf, adjust.method = "fdr",coef = 1)
    >tissue_results <- topTable(fit2, number = Inf, adjust.method = "fdr",coef = 2)
  d. Select for tumor-specific DMPs.

>tissue_not_sig_probes <- tissue_results %>% filter(adj.P.Val >= 0.05 | abs(logFC) < 0.01) %>% rownames()

>tumor_sig <- tumor_results %>% rownames_to_column("probe_id") %>% filter( adj.P.Val < 0.01, abs(logFC) > 0.2, probe_id %in% tissue_not_sig_probes ) %>% arrange(desc(abs(logFC))) %>% slice_head(n = 500) %>% pull(probe_id)

***Note:*** Since plasma cfDNA originates from various tissues throughout the body, we retain only tumor-specific methylation sites by excluding tissue-specific sites that show differences between adjacent normal tissue and normal plasma samples.

**Figure 3:**
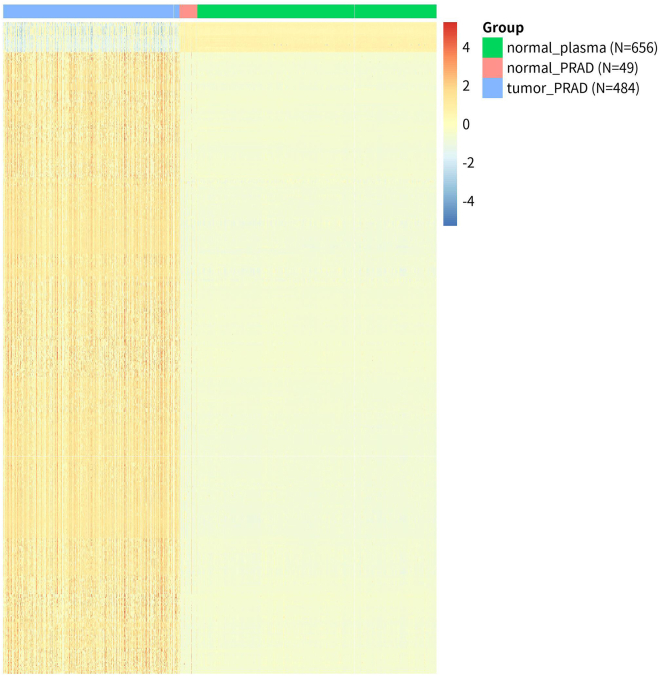
Heatmap of beta values for the top 500 differentially methylated CpG sites

10. Generate a heatmap to illustrate the methylation patterns of the top 500 tumor-specific sites across different sample groups (Figure 3).

>annotation_col <- data.frame(Group = factor(rep(group_names, times = group_sizes), levels = group_names)) group_counts <- table(annotation_col$Group) new_labels <- paste0(names(group_counts), " (N=", group_counts, ")") names(new_labels) <- names(group_counts) annotation_col$Group <- new_labels[as.character(annotation_col$Group)] rownames(annotation_col) <- colnames(beta_matrix)

>pheatmap( as.matrix(beta_matrix[tumor_sig,]), annotation_col = annotation_col, scale = "row", cluster_rows = TRUE, cluster_cols = FALSE, treeheight_row = 0,color = colorRampPalette(rev(brewer.pal(7, "RdYlBu")))(100), show_rownames = FALSE, show_colnames = FALSE,filename = "PRAD_top500.jpg")

11. Save the reference and test sets for Oncoder.

>ref_matrix <- beta_matrix[tumor_sig,which(grepl("tumor",colnames(beta_matrix))|grepl("plasma",colnames(beta_matrix)))]

>ctProstate_Resistant<- matrix_list$ctProstate_Resistant[tumor_sig,]

>ctProstate_Sensitive <- matrix_list$ctProstate_Sensitive[tumor_sig,]

>write.table(ctProstate_Sensitive, file = "test_ctPRAD_Sensitive.tsv", sep = "\t", quote = FALSE)

>write.table(ctProstate_Resistant, file = "test_ctPRAD_Resistant.tsv", sep = "\t",quote = FALSE)

>write.table(ref_matrix, file = "ref_matrix_PRAD.tsv",sep = "\t",quote = FALSE)

>quit()

**CRITICAL:** The reference set comprises 1,140 samples, including 484 prostate tumor samples and 656 plasma samples from healthy donors. The two independent test sets consist of cfDNA methylation matrices derived from 74 AA-sensitive and 107 AA-resistant samples. Ensure that all matrices are saved in the working directory for downstream analysis.

### Implementation of the Oncoder

**Timing: 1–2 h**

**Figure 4:**
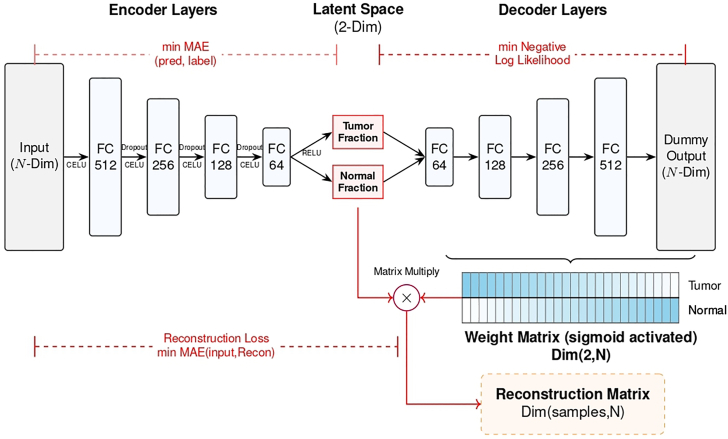
The model architecture of Oncoder The model architecture comprises a deep encoder and a deep decoder. The encoder consists of five non-linear fully connected layers with progressively decreasing dimensions (N, 512, 256, 128, 64, latent dimension), where the input dimension N corresponds to the number of CpG sites selected in Step 9. CELU activation is employed in the hidden layers to ensure robust gradient propagation. The latent dimension is set to 2, representing the inferred tumor and normal fractions. During inference, a ReLU activation is applied to enforce non-negative values of tumor fractions. The symmetric decoder consists of five bias-free linear layers. The effective weight matrix of the decoder represents the learned methylation atlas, with values constrained to the range (0, 1) via a Sigmoid function to correspond to DNA methylation beta-values.

This section includes the details for training Oncoder to monitor the dynamic changes of tumor fractions in AA-sensitive and AA-resistant patients during AA treatment. The model architecture of Oncoder is depicted in Figure 4.

12. Activate the conda environment and import the necessary packages.

>conda activate Oncoder

>cd Oncoder

>python

>import Oncoder

>import torch

>from Oncoder import Autoencoder

>device = torch.device("cuda" if torch.cuda.is_available() else "cpu")

13. Generate 1,000 simulated training samples with labeled tumor fractions by blending tumor tissue and normal plasma methylation profiles at predefined proportions (troubleshooting 4).
  >refpath = './ref_matrix_PRAD.tsv'
  *>t*rain_x, train_y = Oncoder.generate_simulated_data(refpath, prior=[0.8, 0.2], samplenum=1000,random_state=1)

***Note:*** The tumor fraction in real-world plasma cfDNA samples is generally unknown. Hence, we employed a Dirichlet distribution-based approach to generate simulated data as the training set with known tumor fractionlabels.

***Note:*** For details on the parameters and return values of Oncoder’s functions, use the command below:

>help(Oncoder.generate_simulated_data)

This function generates simulated data with tumor fraction labels based on dirichlet or uniform distribution.

Args:

 refpath: the file path of reference data, str

  The file contains a matrix of CpG sites by samples. The index should be CpG sites, and columns should represent sample types (‘plasma_1’, ‘plasma_2’, ... ‘plasma_n’, ‘tumor_1’, ‘tumor_2’, ... ‘tumor_n’). Should not contain null values.

 samplenum: int, optional.

  The number of simulated samples to generate, by default 5000.

 random_state: int, optional

  A seed for the random number generator for reproducibility, by default 1.

 method: {'Dirichlet', 'Uniform'}, optional

 The method to generate tumor fractions, by default ‘Dirichlet’.

Returns:

 x: numpy.ndarray

  The simulated data matrix of shape (samples, cpg_sites).

 y: numpy.ndarray

  The simulated labels matrix of shape (samples, fractions). Column 1: Healthy fractions; Column 2: Tumor fractions.

14. Train the Oncoder model.

>model = Oncoder.train_Oncoder(train_x, train_y, refpath,model_name='ctPRAD', batch_size=128, epochs=256,seed=1,lr=1e-5)

>quit

***Note:*** During model training, the hyperparameter configuration is set as follows: the model is optimized using the Adam optimizer with a learning rate of 1e-4. Dropout (rate = 0.5) is applied after each hidden layer of the encoder to prevent overfitting. The model is trained with a batch size of 128 for 256 epochs. The total loss is a weighted combination of: (1) Comp: the MAE between predicted fractions and ground-truth labels; (2) Recon: the MAE between the input and reconstructed matrices; and (3) Methy-H/Methy-T: the negative log-likelihood of normal and tumor methylation beta values.

**CRITICAL:** Upon completion of training, the model (e.g., ctPRAD.pth) will be saved in the working directory.

**Figure 5:**
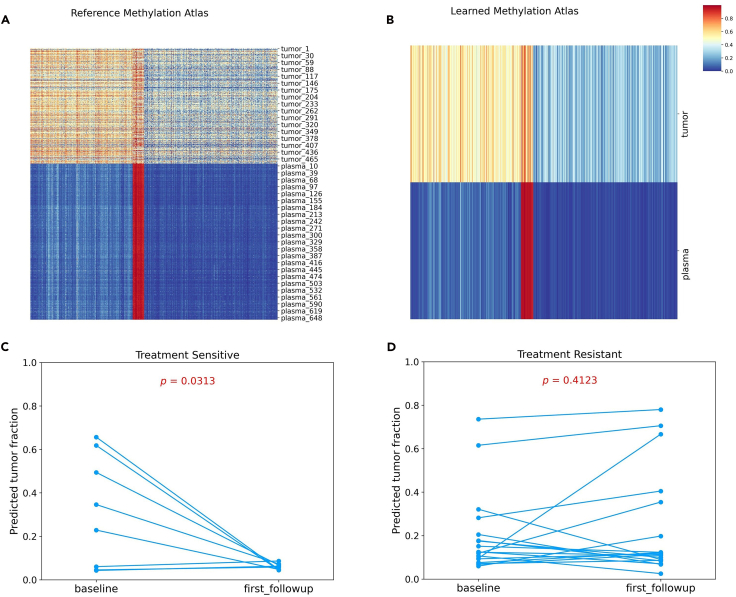
The performance of Oncoder to learn from reference methylation profiles and predict tumor fraction dynamics after AA treatment (A) The reference methylation profiling derived from prostate patients and healthy plasma donors. (B) The methylation atlas of tumor tissues and normal plasma learned by Oncoder. (C and D) Dynamics of predicted tumor fraction in plasma cfDNA from AA-sensitive (C) and AA-resistant (D) patients during AA treatment. Statistical significance is determined by paired t-test.

15. Visualize Oncoder’s performance in learning the methylation atlas (Figures 5A and 5B) and save the learned atlas as a CSV file (Table S1) (troubleshooting 5).

>python plotLearnedAltas.py --file "./train_matrix_PRAD.tsv" --model "./ctPRAD.pth" --save_dir "."

16. Finally, use Oncoder to predict the tumor fraction from AA-sensitive and AA-resistant patient cfDNA methylation data.

>python predict_tumor_fractions.py --file "./test_ctPRAD_Resistant.tsv" --model "./ctPRAD.pth" --outfile "./pred_ctPRAD_Resistant.csv"

>python predict_tumor_fractions.py --file "./test_ctPRAD_Sensitive.tsv" --model "./ctPRAD.pth" --outfile "./pred_ctPRAD_Sensitive.csv"

***Note:*** After running the commands above, CSV files (Tables S2 and S3) recording the tumor and normal fractions for AA-sensitive and AA-resistant patients will be saved.

17. Perform statistical testing and visualize the results (troubleshooting 6):

>python PRAD_statistical_test.py

***Note:*** AA-sensitive patients exhibited a temporary reduction in interindividual variation of their cfDNA methylation profiles during the initial treatment phase.^10^ Therefore, we compared the tumor fraction changes at baseline with those at the first follow-up after AA treatment.

***Note:*** As is shown in Figures 5C and 5D, AA-sensitive patients exhibit a significant decrease in tumor fractions from baseline to the first follow-up (mean change: −0.251 ± 0.264 (SD); paired t-test, p = 0.0313), while AA-resistant patients exhibit no significant difference (mean change: 0.034 ± 0.173 (SD); paired t-test, p = 0.4123).

**CRITICAL:** Upon execution of the above script, JPG files showing the tumor fraction changes will be saved to the working directory. (troubleshooting 5).

### Generalization to other application scenarios

The framework of Oncoder is designed to be data-driven and modular. To expand its application scope, such as adapting the model to other cancer types, the pipeline requires only substituting the tumor tissue datasets (e.g., replacing TCGA-PRAD with TCGA-BLCA in Step 1 for the study of bladder urothelial carcinoma) and re-executing “Differential Methylation Analysis” steps to generate a disease-specific reference set. Furthermore, given that the tumor fractions quantified by Oncoder serve as a direct reflection of ctDNA signal levels in patient plasma, the model’s applicability extends to a broad spectrum of therapeutic modalities, including chemotherapy and immunotherapy, rather than being limited to the specific agent (e.g., abiraterone) evaluated in our case study.

## Expected outcomes

We obtained the training set, test set, and corresponding metadata from the public datasets, and saved them to the working directory. The conversion of the beta-value matrix to an M-value matrix, coupled with the visualization of methylation density (Figures 2A and 2B) and the mean-variance relationship (Figures 2C and 2D), served to validate the greater appropriateness of M-values for subsequent statistical analysis. After performing the differential analysis, a heatmap (Figure 3) was created to display the beta-value differences across the various groups.

The implementation process of the Oncoder model yielded the following results: (1) An autoencoder-based Oncoder model, saved as a “.pth” file in the working directory. (2) JPG files of the reference and learned methylation atlases and a CSV file containing the beta-values of the learned atlas (Figures 5A and 5B; Table S1); (3) CSV files that record the predicted tumor and normal fractions for AA-resistant and AA-sensitive patients, with the first column listing the sample GEO accessions (Tables S2 and S3). (4) JPG files depicting the change in cfDNA tumor fraction from baseline to the first follow-up in AA-treated patients, with statistical significance assessed by paired *t* test (Figures 5C and 5D).

## Quantification and statistical analysis

The methylation beta value represents the percentage of methylation at a CpG site, and is calculated as follows:

$${Beta}_{value}=\frac{M}{M+U+offset\;}$$

Where M and U represent the methylated and unmethylated intensities at a specific probe, and an offset (typically 100) is added to the denominator to regularize the beta value when both methylated and unmethylated probe intensities are low.

The M value does not have a direct biological interpretation. It can be derived from the Beta value using a logit transformation:

$$M_{value}=\log_{2}\left(\frac{Beta}{1-Beta}\right)$$

## Limitations

Our current protocol is constrained by the limited availability of cfDNA methylation samples. Consequently, we rely on tissue-derived data for feature identification. A significant limitation of this approach is that high-performance methylation markers identified in tissues might demonstrate suboptimal performance when applied to cfDNA, due to the distinct biological matrices. Therefore, to enhance the model’s accuracy and clinical applicability, Oncoder should be trained directly on cfDNA-derived markers once a sufficient sample cohort is assembled.

Furthermore, the current version of Oncoder utilizes methylation data profiled using the Illumina HumanMethylation450 BeadChip platform. Our future work will integrate whole-genome bisulfite sequencing (WGBS) data to enable an unbiased discovery of biologically significant methylation patterns, thereby ensuring a substantial enhancement in Oncoder’s resolution and discriminatory power.

## Troubleshooting

### Problem 1

The “GEOquery” package may fail to install because its dependency, “XML”, cannot be compiled, Related to Step 4 before you begin.

### Potential solution

One possible reason is that you installed the libxml2 library from the conda-forge channel. You should install libxml2 from the default channel by running.

>conda install libxml2

### Problem 2

No manifest file exists on the server, Related to Step 1f.

### Potential solution

To upload the manifest file, install a client like FileZilla or XFTP on your Windows PC, then connect to your server and transfer the file.

### Problem 3

It’s possible to get a “Failed to perform HTTP request” error when using the getGEO() function, Related to Step 2a.

### Potential solution

To prevent network timeouts from a busy GEO server or on an unstable connection, increase the timeout duration. Run the following command line in R before using getGEO() function.

>options(timeout = 5000)

### Problem 4

When using the Oncoder.generate_simulated_data function, you may encounter the following issue, Related to Step 13:

KeyError: 'tumor'/'plasma'

### Potential solution

This error occurs when the Oncoder could not identify columns corresponding to ‘tumor’ or ‘plasma’. Please ensure the column names of the input training matrix follow the format tumor_1, tumor_2, ..., tumor_n, plasma_1, plasma_2, ..., plasma_n.

### Problem 5

When using the plotLearnedAlteas.py script, you may encounter the following error, Related to Step 17.

‘ModuleNotFoundError: No module named ‘torch’.

### Potential solution

The Python scripts run using the interpreter in your currently active environment. Please ensure you have activated the required conda environment for Oncoder before executing them.

### Problem 6

When using the PRAD_statistical_test.py script, you may encounter the following issue, Related to Step 17.

FileNotFoundError: [Errno 2] No such file or directory: ‘./pred_ctPRAD_Resistant.csv’ or FileNotFoundError: [Errno 2] No such file or directory: ‘metadata_ctProstate_Resistant.tsv’.

### Potential solution

Prior to executing the script, ensure that the metadata files for AA-resistant and AA-sensitive patients (saved in Step 2e), the tumor fraction prediction files (generated in Step 16), and this script are all located in the same directory.

## Resource availability

### Lead contact

Further information and resource requests should be directed to the lead contact, Xin Li (lixin253@mail.sysu.edu.cn).

### Technical contact

Further technical information and reasonable requests may be directed to and will be fulfilled by the technical contact, Zhenbo Yuan (yuanzhb6@mail2.sysu.edu.cn).

### Materials availability

This study did not generate new unique reagents or materials.

### Data and code availability

• This paper analyzes existing publicly available data, which are listed in the key resources table.
• Code has been deposited to the GitHub repository (https://github.com/ZhenboyYuan/Oncoder). The software has been archived on Zenodo (https://doi.org/10.5281/zenodo.18229358).

## Acknowledgments

We extend our sincere gratitude to the many researchers who generously shared their DNA methylation datasets with the public. This work was supported by the Guangdong Basic and Applied Basic Research Foundation (nos. 2022A1515220204 and 2024A1515013077).

## Author contributions

Y. Yang, Z.Y., and X.L. conceived the project. X.L. secured funding and supervised the work. Z.Y. and Y. Yan wrote the paper with contributions from all authors.

## Declaration of interests

The authors declare no competing interests.

## Supplemental information

Table S1. The methylation profiles learned by Oncoder from the reference methylation atlas, related in step 15

Table S2. Predicted tumor fractions in cfDNA methylation data from AA-resistant patients, related in step 16

Table S3. Predicted tumor fractions in cfDNA methylation data from AA-sensitive patients, related in step 16
